# Neuropeptide S Receptor as an Innovative Therapeutic Target for Parkinson Disease

**DOI:** 10.3390/ph14080775

**Published:** 2021-08-06

**Authors:** Victor A. D. Holanda, Julia J. Didonet, Manara B. B. Costa, Adriano H. do Nascimento Rangel, Edilson D. da Silva, Elaine C. Gavioli

**Affiliations:** 1 Department of Biophysics and Pharmacology, Federal University of Rio Grande do Norte, Natal, RN 59078-900, Brazil; victoricpdf@hotmail.com (V.A.D.H.); juliadidonet@gmail.com (J.J.D.); manarabb@hotmail.com (M.B.B.C.); edsjunior9@gmail.com (E.D.d.S.J.); 2 Agricultural School of Jundiaì, Federal University of Rio Grande do Norte, Macaíba, RN 59280-000, Brazil; adrianohrangel@yahoo.com.br

**Keywords:** Parkinson disease, neuropeptide S, dopamine, oxidative stress, animal models

## Abstract

Parkinson disease (PD) is a neurodegenerative disease mainly characterized by the loss of nigral dopaminergic neurons in the substantia nigra pars compacta. Patients suffering from PD develop severe motor dysfunctions and a myriad of non-motor symptoms. The treatment mainly consists of increasing central dopaminergic neurotransmission and alleviating motor symptoms, thus promoting severe side effects without modifying the disease’s progress. A growing body of evidence suggests a close relationship between neuropeptide S (NPS) and its receptor (NPSR) system in PD: (i) double immunofluorescence labeling studies showed that NPSR is expressed in the nigral tyrosine hydroxylase (TH)-positive neurons; (ii) central administration of NPS increases spontaneous locomotion in naïve rodents; (iii) central administration of NPS ameliorates motor and nonmotor dysfunctions in animal models of PD; (iv) microdialysis studies showed that NPS stimulates dopamine release in naïve and parkinsonian rodents; (v) central injection of NPS decreases oxidative damage to proteins and lipids in the rodent brain; and, (vi) 7 days of central administration of NPS protects from the progressive loss of nigral TH-positive cells in parkinsonian rats. Taken together, the NPS/NPSR system seems to be an emerging therapeutic strategy for alleviating motor and non-motor dysfunctions of PD and, possibly, for slowing disease progress.

## 1. Introduction

Neuropeptide S (NPS) is a 20-amino acid peptide identified via the reverse pharmacology approach as an endogenous ligand of the orphan G protein-coupled receptor, named Neuropeptide S receptor (NPSR) [1,2]. NPSR promotes cellular excitability via G_q_ and G_s_ coupling [3,4,5]; thus, the activation of NPSR stimulates mobilization of intracellular Ca^2+^ and cAMP formation [2,3].

The distribution of NPS and NPSR in the rodent brain has been studied using quantitative RT-PCR and in situ hybridization assays in rat and mouse brains. NPS peptide precursor (ppNPS) mRNA is expressed in very limited brain regions: (i) the principal sensory trigeminal nucleus, (ii) the lateral parabrachial nucleus, and (iii) the locus coeruleus area [6]. Unlike NPS, NPSR mRNA is widely expressed throughout the brain with a higher expression of transcripts in the cortex, olfactory nuclei, thalamus, hypothalamus, amygdala and parahippocampal formation, such as the subiculum [6,7,8]. The expression pattern of the NPSR in the central nervous system suggests that this peptidergic system plays an important role in modulating a variety of physiological functions, such as emotional and sensory processing, arousal, stress, energy homeostasis, endocrine regulation, and learning and memory processes (for a review see: Guerrini et al. [9]). Details about the discovery of this peptide and its receptor, in vivo distribution of this system, biological actions and availability of NPSR ligands can be found in other articles of this Special Issue and in previously published reviews [9,10].

Considering the focus of this review on the NPS system as an innovative therapeutic target to treat Parkinson disease (PD), it is essential to emphasize that NPSR mRNA is expressed in dopaminergic areas, such as the substantia nigra pars compacta and ventral tegmental area [6,7]. Recently, a double immunofluorescence labeling study showed that NPSR is coexpressed with tyrosine hydroxylase (TH)-positive neurons in the substantia nigra [11]. Additionally, noticeable c-Fos expression was observed in TH-positive cells in the substantia nigra after acute icv administration of NPS (10 nmol), but not vehicle [11]. Altogether, these findings suggest a potential role for the NPS system in the modulation of nigral dopaminergic transmission. The first behavioral effect reported after central administration of NPS in mice was hyperlocomotion [2]. Several in vivo studies replicated these stimulatory actions in rodents (see Table 1), and the putative mechanisms were partially described. More recently, studies showing beneficial effects of NPS in counteracting behavioral and neurobiological aspects of PD in rodents have been published [11,12,13,14]. This review aims to briefly present epidemiological and pathophysiological aspects of PD and to discuss the available pharmacotherapy for this neurodegenerative disease. The focus is given in the literature showing in vitro and in vivo findings that candidate NPSR agonists as innovative antiparkinsonian drugs. Additionally, putative mechanisms, by which NPSR activation might be beneficial for Parkinson-related signs and symptoms, will be the object of the present review.

## 2. Pathophysiology and Pharmacotherapy of Parkinson Disease: The Relevance of Innovative Treatments

PD is a progressive degenerative disease manifested by motor and non-motor symptoms [38]. Akinesia/bradykinesia, muscle rigidity, slowness and a resting tremor are the main motor symptoms associated with PD [39]. Non-motor symptoms usually have a gradual development starting years before the first motor symptoms. The prodromal non-motor features of PD include sleep behavior disorder, constipation, urinary dysfunction, loss of smell, excessive daytime sleepiness, orthostatic hypotension and depression [40]. Other common non-motor complications of PD are cognitive deficits, sleep loss, fatigue and anxiety [41].

PD is the second most common neurodegenerative disorder after Alzheimer’s, and it affects over 6 million people worldwide [42]. Possibly due to the neuroprotective actions of estrogens, PD is twice more common in men than women, and the beginning of symptoms starts 2 years earlier in men than women [43]. It is rarely diagnosed in patients under 40 years of age, but the incidence increases with age, and the prevalence of this neurodegenerative disease is around 3% in the population over 80 years [42]. Factors including increasing longevity, declining smoking rates and increasing environmental contamination with by-products of industrialization are contributing to the significant growth in the prevalence of PD [42].

Major motor symptoms in PD are caused by depletion of dopamine in the striatum following a progressive and irreversible degeneration of dopamine-producing neurons in the substantia nigra pars compacta [44,45]. Although the motor symptoms of PD rely on the dopamine deficiency, this disease is associated with other neurotransmitter deficits that are implicated in various motor and non-motor symptoms and signs (for a review see: Schapira et al. [41]). Motor signs and symptoms of PD appear when over 40% of dopaminergic neurons in the substantia nigra are lost, and a decrease in striatal dopamine levels higher than 60% emerges [44]. Histopathological analyses have shown that the loss of dopaminergic neurons is associated with the presence of Lewy bodies, an abnormal aggregation of the protein α-synuclein found inside nerve cells [46,47,48,49].

The therapy for PD is symptomatic, focused on improvement in motor (i.e., resting tremor, rigidity, bradykinesia) and non-motor (i.e., constipation, cognition impairment, mood and anxiety disorders, and sleeping problems) symptoms [38], and it is essentially aimed at increasing dopamine receptor signaling. Considering that dopamine is unable to cross the blood–brain barrier, the dopamine precursor L-3,4-dihydroxyphenylalanine (L-DOPA) is the gold standard of PD therapy [50]. L-DOPA is metabolized to dopamine in the central nervous system by central DOPA-decarboxylase. Therefore, adjuvant therapies to increase the availability of L-DOPA are widely used. Of note, DOPA-decarboxylase and cathecol-o-methyltransferase (COMT) inhibitors are coadministered with L-DOPA to minimize peripheral metabolism [38]. Dopamine is stored in vesicles and then released into the synaptic cleft to stimulate dopamine receptors, leading to a dramatic improvement in the motor symptomatology, mainly akinesia and muscle rigidity [50]. However, important concerns about the L-DOPA therapy must be mentioned: (i) the efficacy of L-DOPA decreases with increasing loss of dopamine neurons, which will no longer allow conversion and sufficient storage of neosynthesized dopamine [51]; (ii) L-DOPA does not stop gradual loss of dopamine neurons and progression of PD [52]; (iii) a large number of side effects are described by PD patients using L-DOPA, including nausea, dry mouth, constipation, medication dose wearing off (“off periods”) and hallucinations [53]; and (iv) some side effects of L-DOPA are irreversible, i.e., dyskinetic disorders (involuntary abnormal movements) [50,51,52].

An alternative pharmacological strategy for PD is represented by dopaminergic agonists that act on the dopamine post-synaptic receptors. Thus, unlike L-DOPA, their pharmacological action is independent of the integrity of dopaminergic neurons [54]. The first class of dopaminergic agonists presents a very high affinity for dopamine D_2_-like receptor and exerts serotonergic and noradrenergic effects [53], which induces heavy side effects, while the new generation is much more specific for the D_2_-like receptor and promotes milder side effects [54]. Other drugs used for alleviating earlier symptoms of PD are inhibitors of monoaminoxidase B (iMAO_B_) and anticholinergic drugs. Generally, dopamine agonists, iMAO_B_ and anticholinergic drugs are used as monotherapy or in association in de novo patients to delay the onset of L-DOPA treatment [51,53,54].

The strategy for PD therapy requires considering the available drugs, and the benefits and risks of each one. The use of L-DOPA results in more functional improvements but has increased dyskinesia [55], while dopamine agonists, iMAO_B_ and anticholinergic drugs are associated with less motor symptoms relief but lower dyskinesia risk [56]. An overall risk of adverse events is observed in these drugs used as alternatives to L-DOPA, such as dopamine agonists, which display a higher risk of impulse-control disorders, sleep attack [57] and withdrawal symptoms [58,59]. It is relevant to mention that no disease-modifying pharmacologic treatments are available [26], and the discovery of innovative drugs to relieve symptomatology of PD and prevent neural death is urgently required.

## 3. Behavioral Effects of Neuropeptide S: Implications for Parkinson Disease

Several biological functions have been reported since the discovery of the NPS/NPSR system in 2004 [2]. Some of them are able to counteract motor and non-motor signs and symptoms of PD, such as the stimulatory effects on locomotion [2,15,18,23,26,60], facilitation of learning and memory processes [61,62,63,64], relief of anxiety (for a review see: Pape et al. [65]) and positive effects on mood [66].

The hyperlocomotor effects of NPS are robust and have been replicated by several laboratories around the world (Table 1). The pioneer study of Xu and collaborators [2] showed that central administration of NPS increases locomotor activity in mice at lower doses (0.1 nmol), and this effect lasts for about 1 h. These findings were largely replicated [4,19,20,26,27,67] and extended to rats [33,34,35,36]. Distinct from classical psychostimulant drugs (i.e., amphetamines), repeated NPS injections did not lead to locomotor sensitization [34]. Recently, stimulant effects of NPS were demonstrated to be due to the selective activation of NPSR. Of note, different NPSR antagonists (SHA-68; RTI-118; [D-Val^5^]NPS; [(t)Bu-d-Gly(5)]NPS) were inactive, per se, but were able to counteract the NPS-induced hyperlocomotion [5,15,16,68,69]. In addition, central administration of NPS did not change the locomotor activity in knockout mice for the NPSR receptor gene (NPSR (−/−)) [28,29]. Genetic studies showed that NPSR (−/−) did not differ from wild-type controls in the spontaneous locomotion [28,29,30,31], while a significant reduction in the cumulative distance moved was observed in the dark phase [30]. These findings demonstrate that NPS effects are exclusively due to NPSR activation and suggest that the endogenous system does not exert a tonic control on spontaneous locomotion in mice.

**Figure 1:**
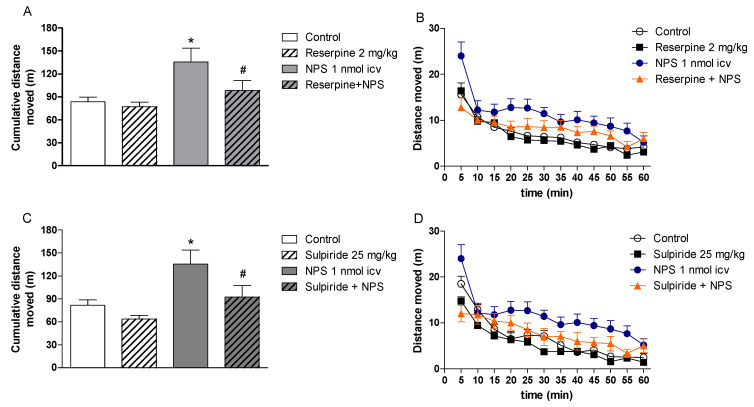
The hyperlocomotor effects of Neuropeptide S (NPS) are impaired in mice pretreated with drugs able to block dopaminergic transmission. Pretreatment with (**A**,**B**) reserpine 2 mg/kg (sc, 24 h before NPS), an inhibitor of vesicular uptake of catecholamines and 5-HT, and (**C**,**D**) sulpiride 25 mg/kg (ip, 45 min before NPS), a D_2_-like receptor antagonist, prevented the hyperlocomotor effects of NPS 1 nmol (icv, 15 min prior to the test) in male Swiss mice assessed in the open field (40 × 40 × 40 cm) test for 60 min. Data are presented as cumulative distance moved (m) and distance moved (m) in blocks of 5 min. * *p* < 0.05 vs. Control; # *p* < 0.05 vs. NPS. ANOVA, Dunnett’s post hoc test ((**A**); F (3,50) = 8.46; *p* < 0.05; (**C**); F (3,43) = 9.93; *p* < 0.05).

A growing body of evidence suggests that NPS-induced hyperlocomotion is mediated by dopaminergic transmission. Firstly, immunohistochemical studies showed that NPSR is moderately expressed in dopaminergic nuclei, such as the ventral tegmental area, basal ganglia, and substantia nigra pars compacta in rats [6,8] and mice [7]. Additionally, unpublished data from our research group demonstrated that pretreatment with reserpine (Figure 1A,B), an inhibitor of vesicular uptake of catecholamines and 5-HT, prevented the hyperlocomotor effects of icv administration of NPS. Similar results were observed with systemic pretreatment with sulpiride, a D_2_-like receptor antagonist, in NPS treated mice (Figure 1C,D). In agreement with these findings, Mochizuki et al. [27] demonstrated the participation of the mesolimbic dopaminergic pathway in NPS-induced hyperlocomotion. Intracranial injection of NPS in the ventral tegmental area, significantly and dose-dependently, increased motor activity in rats [27]. This in vivo effect was dose-dependently inhibited by pre-administration of sulpiride into the shell of the nucleus accumbens [27].

More recently, a double immunofluorescence labeling study showed that NPSR is intensely coexpressed with TH-positive neurons in the substantia nigra [11], thus reinforcing the modulatory role of NPSR signaling in controlling dopamine transmission in the basal ganglia. The basal ganglia are particularly relevant for motor programming and execution [70]. Interestingly, NPS infused into the substantia nigra and globus pallidus dose-dependently increased mouse locomotor activity [15]. SHA-68, an antagonist of NPSR, when systemically injected, blocked the stimulant effects of NPS in both brain areas [15]. Taken together, preclinical findings support the hypothesis that dopaminergic transmission from mesolimbic and nigral pathways, possibly involving D_2_-like receptor signaling, mediates the locomotor effects of NPS.

Behavioral studies using the neurotoxin 6-hydroxydopamine (6-OHDA) were performed to mimic PD dysfunctions in rodents and investigate the potential therapeutic effects of NPS (Table 2). A pilot study published in 2014 showed that central injection of NPS restored icv administration of 6-OHDA-induced motor deficits in mice [12]. Similar to the positive control L-DOPA, NPS dose-dependently attenuated motor impairments assessed in the rotarod test in mice [12]. The effects of NPS in counteracting motor dysfunction of PD were recently confirmed by Bülbül et al. [11] and Sinen et al. [14] in rats. For instance, acute and repeated icv injection of NPS (10 and 1 nmol, respectively) significantly improved 6-OHDA-induced hypolocomotion and motor incoordination [11,14] as well as restored reduction in nigral dopamine release [11].

The beneficial effects of NPS were also extended to non-motor symptoms of PD. Recently, a study showed that chronic NPS (1 nmol, once a day for 7 consecutive days) administration also alleviates 6-OHDA-induced non-motor behaviors in rats, such as working memory deficits, anxiety and depression-like behaviors [13], as well as facilitating solid gastric emptying in a parkinsonian animal [14]. Importantly, expression of NPSR protein was detected in gastro-projecting cells in the dorsal motor nucleus of vagus, thus explaining, in part, the gastric effects of NPS in a PD rat model [14]. These preclinical findings support the hypothesis that NPS could be a candidate for the treatment of motor and non-motor signs and symptoms of PD. Unfortunately, these effects of NPS in PD are short-lived, limited to 60 min, and require central injections. An alternative to the central administration of peptides, such as NPS, is the intranasal route of administration. Of note, intranasal administration of NPS reduced anxiety-like behaviors and alertness in rats [24,71]. This strategy therefore appears to be a useful alternative route of administration for treatment of central nervous system disorders, including PD [72]. NPSR nonpeptide agonists are still not available, but studies are in progress for developing these compounds [10]. Future studies using NPS intranasal formulations, and/or small molecules acting as NPSR agonists, are needed for further validation of antiparkinsonian effects and possibly for performing clinical studies.

## 4. Putative Mechanisms by Which Neuropeptide S Alleviates Parkinson Disease Signs and Symptoms

**Figure 2:**
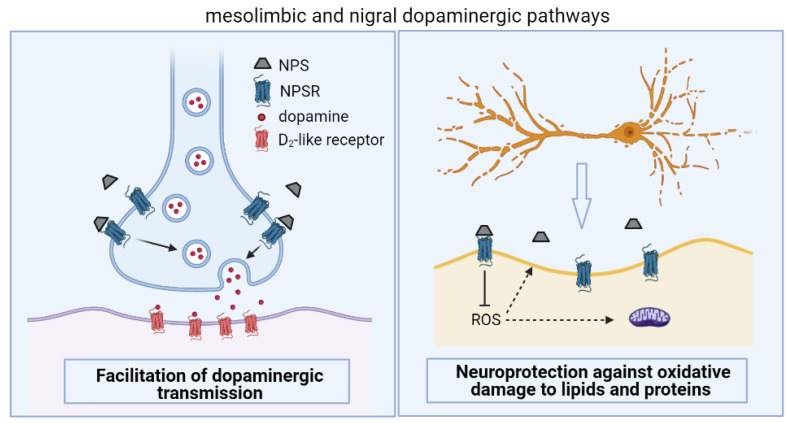
Putative mechanisms by which Neuropeptide S (NPS), activating Neuropeptide S receptor (NPSR), alleviates Parkinson Disease (PD)-induced motor and non-motor dysfunctions. NPS increases dopaminergic transmission in mesolimbic and nigral dopaminergic pathways by facilitating the dopamine transmission (**left panel**). NPS promotes neuroprotective effects on dopaminergic neurons possibly by counteracting reactive oxidative species (ROS)-induced damage to structural lipids and proteins (**right panel**).

Considering the aforementioned neurobiological changes that occur in PD, and the beneficial effects of NPS in counterbalancing motor and non-motor dysfunctions of this degenerative disease, this section will discuss the putative mechanisms mediating the effects of NPS (Figure 2). A vast body of literature shows that neurodegeneration of dopamine neurons in the substantia nigra pars compacta leads to the reduction in dopamine in basal ganglia, consequently contributing to motor symptoms of PD [45]. Therefore, the first hypothesis of the mechanism by which NPS alleviates PD dysfunctions is by facilitating dopaminergic transmission. The first evidence that corroborates this hypothesis comes from in vivo microdialysis studies in freely moving rats, in which NPS injected into the ventral tegmental area elevates extracellular dopamine metabolites (3,4-dihydroxy-phenyl acetic acid and homovanillic acid) in the nucleus accumbens [27]. Similar findings were observed when NPS was injected into the lateral ventricle, and the extracellular levels of dopamine and its metabolites were measured in the medial prefrontal cortex [73]. Animal models of PD give further support to the NPS modulatory effects on dopaminergic transmission. For instance, in vivo microdialysis studies showed that acute and 7 days of NPS central administration significantly restored dopamine levels in the substantia nigra [11] and hippocampus [13], but not in the striatum [13], in parkinsonian rats. Therefore, beneficial effects of NPS on PD motor and non-motor dysfunctions may be due to the stimulation of NPSR expressed by dopaminergic neurons in the nigral and mesolimbic pathways.

Another possible mechanism by which NPS exerts beneficial effects on PD symptomatology is due to putative neuroprotective properties. Castro et al. [18,21] showed reduced carbonylated proteins and lipid peroxidation in a mouse brain after NPS central administration. The protective effects of NPS against oxidative damage to macromolecules were observed when mice received pentylenetetrazole, a GABA_A_ antagonist used for inducing seizures and damage to macromolecules [74]. In parkinsonian rats, 7 days of central treatment with NPS (1 nmol, once a day for 7 consecutive days), given after 6-OHDA administration, prevented the degeneration of TH-positive nigral neurons [11,13,14]. It is relevant to mention that other neurotransmitter systems are also dysfunctional in PD, including serotonin, acetylcholine and noradrenaline systems [38]. Chronic treatment with centrally injected NPS prevented the loss of cholinergic neurons in vagus nucleus, which could possibly be a mechanism for blunting the 6-OHDA-induced effects on gastric cholinergic transmission [14]. These effects of NPS can possibly involve protection against oxidative damage to lipids and proteins. This hypothesis is supported by the fact that chronic NPS administration in PD rats reduced the expression of 4-hydroxynonenal (4-HNE), a product from lipid peroxidation, in the substantia nigra [11].

The mechanism of nigral neurodegeneration in PD is still not completely understood. However, it has been shown that 90% of α-synuclein in Lewy bodies is phosphorylated at serine 129 in a PD brain, while it is around 5% in a normal brain [75]. The accumulation of Ser^129^-phosphorylated α-synuclein leads to the formation of Lewy bodies and dopaminergic neurodegeneration in the pathogenesis of PD [76]. Using an immunohistochemistry approach, it has shown that repeated administration of NPS attenuated 6-OHDA-induced Ser^129^-phosphorylation in α-synuclein both in nigral neurons and dorsal motor nucleus of the vagus [14]. Several factors have been considered through time, such as mitochondrial dysfunction, α-synuclein toxicity and neuroinflammation, to all of which oxidative stress can be seen as a common denominator [77]. Further studies are needed to investigate the mechanisms by which NPS (and NPSR agonists) evokes neuroprotective effects in PD. If confirmed, NPSR agonists can be a completely innovative therapeutic strategy to prevent PD neurodegeneration.

The adenosinergic system could be another mechanism by which NPS mediates antiparkinsonian effects. In fact, adenosine A_2A_ receptor antagonists, including caffeine, are reported to be innovative therapeutic strategies for the treatment of behavioral symptoms and neurodegeneration of PD [78,79]. A parallel between caffeine and NPS effects has already been drawn in the literature. For instance, NPS and caffeine induce similar effects on locomotion [26], modulation of wakefulness states [2,26] and food intake [33,37,80,81] Additionally, interaction between adenosinergic and NPS-NPSR receptor systems has been postulated in the modulation of locomotion [22] and nociception [82]. Furthermore, administration of caffeine, an adenosine A_2A_ receptor antagonist, downregulated NPS brain levels in rats [83]. Recently, a complex interaction between NPSR genotype, caffeine administration and startle responses in humans was postulated, thus linking the NPS and adenosine systems in the modulation of anxiety [84]. Considering the relevance of the adenosinergic system in the pathophysiology of PD, and the interplay between NPS and adenosinergic systems, further studies are needed to investigate the participation of adenosine receptors in the antiparkinsonian effects of NPS.

## 5. General Conclusions and Future Perspectives

The beneficial effects of NPS as an emerging alternative for treating signs and symptoms of PD have been discussed. Using animal models of PD, NPS significantly improved motor and non-motor dysfunctions. Dopaminergic transmission seems to be mediating some of the positive actions of NPS on PD. Additionally, in parkinsonian rats, 7 days of NPS administration protected them from degeneration of nigral dopaminergic neurons possibly by reducing oxidative damage to lipids and proteins, and by attenuating phosphorylated α-synuclein. Therefore, preclinical studies using distinct animal models of PD aiming to investigate the long-term effects of NPSR agonists as monotherapy or as an adjuvant strategy to treat PD are urgently needed.

Considering that in vivo assays were performed in NPS central injected rodents, further studies aiming to evaluate the effects of NPS using the intranasal route and small-molecule NPSR agonists are necessary for extending available preclinical findings and possibly starting clinical studies. If confirmed, NPSR selective agonists can be the first disease-modifying approach able to simultaneously treat PD dysfunctions and counteract the progression of the disease.

## Figures and Tables

**Table 1:** Effects of Neuropeptide S (NPS) on spontaneous locomotion and exploration of animals in novel and familiar environments.

Animal Specie (Condition)	Active Doses and Route of Administration	Experimental Assay	Behavioral Effects	References
mouse (naïve)	0.1–1 nmol (icv)	open field test	↑ total distance moved; ↑ number of rearings	[2,4,5,15,16,17]
			↑ total distance moved	[7,18,19,20,21,22]
	0.45 nM, 2 µL (icv)		↑ ambulatory activity	[23]
	7, 14, and 28 nmol (intranasal)		no effects on distance moved	[24]
	1, 10 and 50 nmol (icv)	home cage locomotion	no effects on spentaneous locomotion	[25]
	0.1–1 nmol (icv)	activity cage	↑ number of pulses in naïve and habituated mouse	[26]
	0.05–0.5 nmol (intra-VTA)		↑ total distance moved; ↑ number of rearings	[27]
mouse (diazepam-treated)	0.1–1 nmol (icv)	activity cage	↑ number of pulses	[26]
NPSR (−/−) mouse	0.1 nmol (icv)	open field test	no effects on locomotion	[28]
	-		no differences compared to wild type mice	[28,29,30,31]
	-		↓ distance moved in the dark phase	[30]
NPS (−/−) and NPS (+/−) precursor mouse	-	open field test	↓ cumulative distance moved	[32]
rat (naïve)	0.1–10 nmol (icv)	open field test	↑ horizontal activity; ↑ rearing activity	[33,34,35]
	0.2 nmol (into MeA)		↑ total distance moved	[36]
chicken	0.625 μg (icv)	open field test	↑ total distance moved	[37]

Abbreviations: icv: intracerebroventricular; MeA: medial amygdala; mfb: medial forebrain bundle; NPSR: neuropeptide S receptor; VTA: ventral tegmental area.

**Table 2:** Behavioral and neurobiological effects of Neuropeptide S (NPS) treatment in animal models of Parkinson Disease (PD).

Animal Model of PD	Specie (Strain, Weight, Sex)	NPS Treatment (Dose, Route, etc)	Effects of NPS	References
6-OHDA, icv, 50 µg/2 µL	mouse (Swiss, 28–35 g, female)	0.1 and 1 nmol (icv), 15 min before behavioral test	Acute NPS reversed 6-OHDA-induced motor incoordination	[12]
6-OHDA, into right MFB, 12 µg/3 µL	rat (Wistar, 250–300 g, male)	1 nmol (icv), 7 consecutive days after 6-OHDA 10 nmol (icv), acutely, on 7th day after 6-OHDA	Acute NPS reversed 6-OHDA-induced motor incoordination, locomotor deficits and catalepsy; Repeated NPS reversed 6-OHDA-induced hypolocomotion and motor incoordination; Acute and repeated NPS restored 6-OHDA-induced dopamine concentrations deficits in nigral microdialysates; Repeated NPS partially attenuated 6-OHDA-induced degeneration on nigral TH immunoreactive cells; Repeated NPS attenuated 6-OHDA-induced nigral 4-HNE immunoreactivity	[11]
6-OHDA, into right MFB, 12 µg/3 µL	rat (Wistar, 250–300 g, male)	1 nmol (icv), 7 consecutive days after 6-OHDA	Repeated NPS restored 6-OHDA-induced reference and working memory deficits in the radial maze task; Repeated NPS restored 6-OHDA-induced anhedonic behavior in the sucrose preference test; Repeated NPS reduced 6-OHDA-induced dopamine deficits in the hippocampus, but not in the striatum; Repeated NPS partially attenuated 6-OHDA-induced degeneration on nigral TH immunoreactive cells	[13]
6-OHDA, into right MFB, 12 µg/3 µL	rat (Wistar, 250–300 g, male)	1 nmol (icv), 7 consecutive days after 6-OHDA	Repeated NPS reversed 6-OHDA-induced locomotor deficits in an unfamiliar environment; Repeated NPS partially attenuated 6-OHDA-induced degeneration on nigral TH immunoreactive cells; Repeated NPS reversed 6-OHDA-induced changes in gastric empty; Repeated NPS prevented 6-OHDA-induced expression of phosphorylated α-synuclein in substantia nigra, dorsal motor nucleus of the vagus and hypoglossal nucleus; Repeated NPS attenuated 6-OHDA-induced degeneration of the cholinergic neurons of the dorsal motor nucleus of the vagus	[14]

Abbreviations: 4-HNE: 4-hydroxynonenal; 6-OHDA: 6-hydroxydopamine; icv: intracerebroventricular; MFB: medial forebrain bundle; TH: tyrosine-hydroxylase.

## Data Availability

Not applicable.
